# Anti-Leukemic Properties of Curcumin on Acute Lymphoblastic Leukemia: A Systematic Review

**DOI:** 10.3390/biology15030258

**Published:** 2026-01-30

**Authors:** Teck Chee Soh, Ying Hui Tan, Pen Han Heng, Faizatul Isyraqiah, Rakesh Naidu, Kok-Lun Pang

**Affiliations:** 1 Jeffrey Cheah School of Medicine and Health Sciences, Monash University Malaysia, Bandar Sunway, Subang Jaya 47500, Malaysia; tsoh0005@student.monash.edu (T.C.S.); tanyinghui01@gmail.com (Y.H.T.); phen0017@student.monash.edu (P.H.H.); faizatul.muhammad@monash.edu (F.I.); rakesh.naidu@monash.edu (R.N.); 2 Monash Medical Centre, Monash Health, Clayton, Melbourne, VIC 3168, Australia

**Keywords:** autophagy, apoptosis, Bcr-Abl, DNA damage, MDR1, oxidative stress, WT1

## Abstract

Background: Acute lymphoblastic leukemia (ALL) is a hematological malignancy characterised by uncontrolled proliferation of lymphoid cells. Despite improved outcomes with modern chemotherapy, treatment resistance and adverse effects remain major clinical challenges. Curcumin, a natural compound from *Curcuma longa*, has shown anticancer potential in multiple malignancies, including leukemia. This systematic review aims to summarise preclinical and clinical evidence on the anti-leukemic effects and mechanisms of action of curcumin in ALL. Methods: A literature search was conducted in August 2025 across PubMed, Scopus, Ovid MEDLINE, and Web of Science according to Preferred Reporting Items for Systematic Reviews and Meta-Analyses 2020 guidelines. Primary research involving in vitro, in vivo, and human studies examining curcumin’s anti-leukemic effects on ALL were included. Of the 2034 records screened, 26 articles met the inclusion and exclusion criteria. Results: Curcumin inhibited proliferation and induced cytotoxicity and apoptosis in ALL cells via reactive oxygen species generation, DNA damage, mitochondrial dysfunction, and caspase activation. It also inhibited the Janus kinase/signal transducer and activator of transcription (JAK/STAT) and phosphoinositol-3 kinase/protein kinase B (PI3K/AKT) signalling, downregulated breakpoint cluster region-Abelson (*BCR-ABL*), Wilms tumor 1 (*WT1*), and Multidrug resistance 1 (*MDR1*) mRNAs, and induced ceramide accumulation and autophagy. In vivo evidence was limited, and no human studies were identified. Conclusions: Curcumin exerts multi-targeted anti-leukemic effects in ALL. Clinical translation is constrained by its poor bioavailability and limited clinical data. Future research should focus on improving the bioavailability of curcumin via chemical or pharmaceutical modification, as well as conducting well-designed clinical trials.

## Simple Summary

Acute lymphoblastic leukemia (ALL) is the most common cancer in children, where immature white blood cells grow uncontrollably. While chemotherapy can treat it, side effects and resistance are common. Curcumin, a natural substance found in turmeric, has shown promising anticancer effects. This systematic review scientifically summarises the anti-leukemic effects of curcumin from cell lines, animal and human. Curcumin works through multiple ways, including damaging cancer cell DNA, causing stress inside the cells, blocking growth signals, and some additional mechanisms in causing suicidal cell death in ALL cells. However, studies in animals are very limited, and there is no solid evidence from human trials yet. One major challenge is that curcumin is not easily absorbed in the body, which makes it harder to use as a medicine. More research is needed to improve how curcumin is delivered in the body and to test its safety and effectiveness in people with ALL.

## 1. Introduction

Acute lymphoblastic leukemia (ALL), also known as acute lymphocytic leukemia, is a haematological malignancy that arises from the lymphoid cell lineage [1]. This disease results from genetic aberrations or mutations in the differentiation pathway of lymphoid progenitor cells, leading to uncontrolled proliferation and diminished apoptosis of immature lymphoblasts in the bone marrow [1]. Consequently, the high number of circulating blast cells replaces normal bone marrow cells and may infiltrate the peripheral blood circulation and other organs. According to the National Comprehensive Cancer Network, a diagnosis of ALL is made when lymphoblasts constitute ≥ 20% of nucleated bone marrow cells [2]. ALL exhibits a bimodal age distribution, with the first incidence peak in childhood and a second peak among adults older than 50 years [3,4]. According to the Global Burden of Disease 2023 [5], the age-standardised incidence of ALL was 1.71 per 100,000 population worldwide. In the United States (US), approximately 6100 new cases and 1400 deaths are projected for 2025 [3]. Based on the Surveillance, Epidemiology and End Results Program (SEER) data, the overall 5-year survival rate in the US is around 72.6% [3]. Children with ALL usually respond well to the standard treatment, with a 5-year survival rate of more than 90% [6]. However, the prognosis worsens with age, with 5-year survival rates of 74% for adolescents, 43–59% for adults, and below 30% for elderly patients [7].

Treatment for ALL involves a range of therapies, including systemic chemotherapy, intrathecal therapy, tyrosine kinase inhibitors (TKIs), allogeneic hematopoietic stem cell transplantation, targeted immunotherapy drugs, radiation therapy, and supportive care, tailored to the patient’s clinical condition [8]. Despite their efficacy, these treatments are commonly associated with undesirable and potentially life-threatening adverse effects, including diarrhoea, nausea and vomiting, neutropenia, anaemia, and neurotoxicity [2]. For instance, rapid destruction of leukemic cells may trigger tumour lysis syndrome [9]. In addition to gastrointestinal toxicities, chemotherapy and immunotherapy significantly increase the risk of infections, thrombosis, neuropathies, hepatotoxicity, anaphylaxis, cytokine release syndrome, and hypothalamic–pituitary–adrenal axis dysregulation [10]. Furthermore, radiotherapy is associated with long-term neurocognitive deficits, endocrinopathies, impaired growth, and secondary central nervous system malignancies [10]. Vulnerable populations, such as infants, adolescents, the elderly, and patients with Down Syndrome, experience higher risks of treatment-related toxicity and mortality [10]. Managing older ALL patients remains particularly challenging, as conventional treatments are often poorly tolerated and less effective [11]. Although treatment outcomes are favourable in younger patients, treatment-related toxicity frequently leads to noncompliance, contributing to relapse and the development of multidrug resistance (MDR). Relapse occurs in approximately 10% of children and up to 50% of adults who achieved complete remission [12]. While stem cell transplantation can provide durable remission or cure, it is associated with other risks, and identification of a suitable donor may be time-consuming [10]. These limitations highlight the urgent need for safer and more effective therapeutic strategies for ALL.

**Figure 1:**
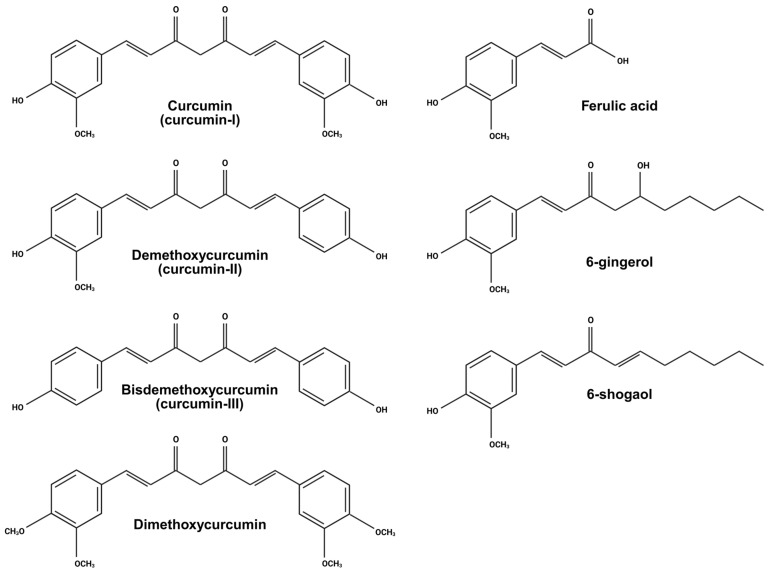
Chemical structure of curcumin and its analogues. Created in BioRender. Pang, K. (2026) https://BioRender.com/v3i4e9t, accessed on 25 December 2025.

Natural compounds have long been investigated for their therapeutic potential against malignant diseases due to their broad spectrum of pharmacological activities. Curcumin or curcumin-I, scientifically known as diferuloylmethane, is a well-recognised bioactive compound derived from turmeric and exhibits diverse medicinal properties (Figure 1). Isolated from the rhizomes of the *Curcuma longa* plant, curcumin is a natural yellow pigment traditionally used in cooking, as well as for colouring, antiseptic, and cosmetic purposes. As a polyphenolic molecule, curcumin has been reported to possess anti-inflammatory, antioxidative, hypoglycaemic, antimicrobial, neuroprotective, and anticancer effects [13]. Accumulated evidence has demonstrated that it has potential efficacy against a variety of cancers, including gastric, skin, prostate, breast, colon, lung, ovarian, and liver cancers [14]. Curcumin is well-tolerated and generally safe for consumption at doses as high as 12 g/day [14]. Despite several related reviews on the anti-leukemic effects of curcumin being reported [15,16,17], to our best knowledge, a systematic review that summarises its anti-leukemic effects, especially on ALL, is not available. Therefore, this systematic review aims to summarise the anti-leukemic properties of curcumin based on in vitro, in vivo, and human studies.

## 2. Methodology

### 2.1. Literature Search

This systematic review was conducted in accordance with the Preferred Reporting Items for Systematic Review and Meta-analyses (PRISMA) 2020 guidelines and checklist (Supplementary Table S1) [18]. We searched the literature using four electronic databases, including PubMed, Web of Science (WoS), Scopus, and Ovid MEDLINE until August 2025 (Supplementary Table S2). The following keywords and Boolean operators were used: ((curcumin) OR (“natural yellow 3”) OR (diferuloylmethane) OR (“turmeric yellow”)) AND ((leukaem*) OR (leukem*)). The curcumin-related terms were selected based on PubMed Medical Subject Heading (MeSH) terms. Additionally, a manual literature search was conducted to identify additional records from the reference lists of included articles. The literature searching protocol was registered and publicly available on the Open Science Framework at https://osf.io/jdp64/ (accessed on 25 December 2025).

**Figure 2:**
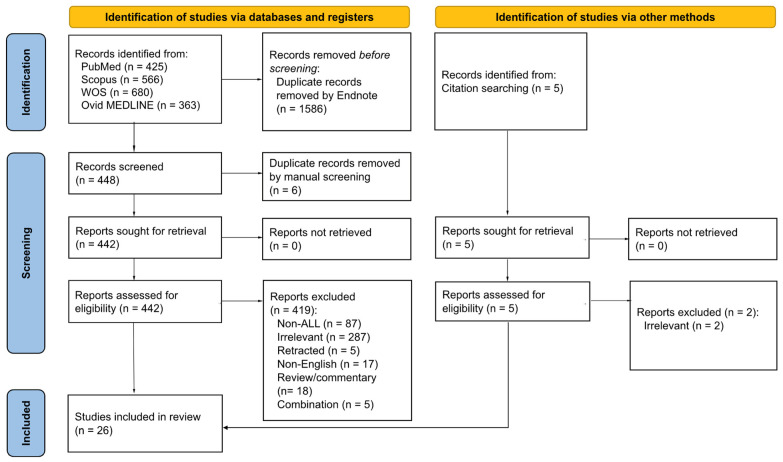
PRISMA flow chart of this systematic literature search.

The records from all databases were first imported into EndNote 21 software as a library. Duplicates were removed using the software’s tool and manually reconfirmed. The remaining records were first screened by three reviewers independently for eligibility based on their titles and abstracts, followed by a full text review. Any discrepancies were resolved through discussions among all reviewers. Research articles reporting the anti-leukemic effects of curcumin, specifically on ALL, were included, regardless of whether the studies were in vitro, in vivo, or human studies. There were no restrictions on the year of publication. However, we excluded studies that were: (1) written in a non-English language; (2) lacked primary or full data (e.g., review articles, meta-analyses, reports, books, book chapters, commentary articles, and conference/proceeding papers); (3) published as a preprint; (4) retracted; or (5) focused on curcumin analogues, Curcuma extracts, or combinations with other non-specific bioactive compounds, without curcumin data. After finalising the articles, three reviewers extracted essential information from the included articles and summarised it in Table 1 independently, which includes authors’ names, publication years, ALL models, curcumin details, treatment conditions, findings, and critical appraisal score. A PRISMA flow chart summarising our procedures for article identification, screening, and final inclusion of articles is presented in Figure 2.

### 2.2. Critical Appraisal

Two reviewers independently assessed the quality of the included articles using a modified OHAT risk of bias tool for in vitro studies and the OHAT risk of bias tool for animal studies [47,48,49]. We evaluated each domain accordingly, with one question per domain. Full compliance and direct evidence of a low risk of bias in one domain were recorded as “*Definitely low risk of bias* (+ +)”. Mild deviations or indirect evidence of a low risk were marked as “*Probably low risk of bias* (+)”. Insufficient information on relevant risk of bias or indirect evidence of a high risk of bias was recorded as “*Probably high risk* (−)”, and lastly, “*Definitely high risk* (− −)” was used for direct evidence of high-risk bias practices. Overall appraisal utilised a 3-level tiering of quality according to previous studies [48,50]. Studies classified as Tier 1 were considered high-quality articles with >50% of *Definitely* and/or *Probably Low Risk*; Tier 2 as moderate quality; while Tier 3 were poor-quality articles with >50% of *Definitely* and/or *Probably High Risk*. Any disagreement between authors was resolved by discussion. The OHAT tier is included in Table 1, and detailed information is shown in Supplementary Tables S3 and S4.

## 3. Results

### 3.1. Characteristics of Included Articles

Our literature search across four databases retrieved 2034 articles: 425 records from PubMed, 566 records from Scopus, 680 records from WoS, and 363 records from Ovid MEDLINE. After removing duplicates using EndNote software and manual check, 442 records were selected for further screening and full-text eligibility assessment. Of these, 419 records were excluded from the final analysis for the following reasons: irrelevant content (*n* = 287), non-ALL studies (*n* = 87), retracted articles (*n* = 5), non-English articles (*n* = 17), review or commentary articles (*n* = 18), and combinational studies lacking anti-leukemic data specific to curcumin (*n* = 5). A total of 26 articles were included to be relevant in this systematic review, including 3 records identified through reference searching.

In this systematic review, most of the included studies used immortalised T lymphoid ALL-derived cell lines, including Jurkat cells [19,20,21,24,30,31,32,40], CCRF-CEM cells [20,24,26,29,30,34,38,45], MOLT-4 cells [24,27,32,41,44], HSB2 cells [24], KOPT-K1 [42], DND-41 [42], and TALL-1 cells [42]. Despite B lymphoid ALL being the major subtype of ALL in both children and adults [51], relevant studies on B-ALL in this systematic review were far fewer than T-ALL. The immortalised B-ALL cell lines include human childhood REH cells [32,35,36,39], childhood SUP-B15 cells [37,39,45], childhood 697 cells [39], and adult RS4;11 cells [35,39]. Doxorubicin-resistant CCRF-CEM cells with overexpressed P-glycoprotein (P-gp; also known as MDR1) were also being studied [38]. Virus-transformed models such as human EBV-related Burkitt lymphoma lymphoblast-like Raji cells [33] and HTLV-1 transformed T cell leukemia cell lines (MT-2, HuT-102 and SLB-1 cells) [25] were also studied. Only four studies used primary patient-derived ALL cells isolated from the peripheral blood [41] or bone marrow [22,23,45] for ex vivo short-term exposure to curcumin. Nevertheless, the cell concentration used in treatment varied within the same studies and across studies. The majority of the included studies were inconsistent in treatment conditions [19,29,32,33,41] or did not disclose this information for some or all experiments [21,22,23,24,26,27,34,35,37,39,40,42,45]. Some studies used cell number instead of cell concentration [20,24,26,29,30,32,33,35,36,38,41,45]. Rajasingh et al. and Koszałka et al. consistently used 2.5 and 3 × 10^5^ cells/mL in their studies [25,44]. Only two studies used ALL xenograft animal models, which included BALB/c null mice with intravenous injection of 10^7^ human B-ALL SUP-B15 cells [45] and NOD/SCID mice with intravenous injection of 5 × 10^6^ human B-ALL SEM cells [46]. To date, there have been no reports of curcumin treatment in patients with ALL.

Most studies sourced curcumin from Sigma [20,21,22,24,27,29,30,32,34,35,36,37,39,40,44,45], though some were obtained from Merck [19], Invitrogen^TM^ Life Technology [23], Calbiochem [25], Cayman Chemical [31,46], Acros Organics [33], and Fluka AG [38]. Two studies purified [27] or synthesised curcumin by themselves [42]. Two studies did not disclose the source or manufacturer of curcumin [26,41]. Anuchapreeda et al. [22,23,27], Kong et al. [29], Olivas-Aguirre et al. [40], Koszałka et al. [44], and Zunino et al. [46] stated the purity of curcumin used, which ranged from ≥65% to 99%. Anuchapreeda reported that commercial-grade and self-extracted curcumin from turmeric powder contain 77% curcumin (or curcumin-I), 17% demethoxycurcumin (or curcumin-II), and 3% bisdemethoxycurcumin (or curcumin-III) [22,23,27]. Piwocka et al. and Khar et al. dissolved curcumin in ethanol [19,20], and most studies used dimethyl sulfoxide to dissolve curcumin [29,30,31,34,35,37,39,41,44,45,46]. The others did not disclose how they dissolved curcumin. In addition, some studies disclosed the maximum concentration of vehicle used in treatment [20,29,31,39,40,44], the concentration of stock solution [29,30,34,37,40,41,44,45], and/or storage conditions for stock solution [29,30,37,41,45,46]. For in vitro studies, the tested concentrations of curcumin were up to 100 μM for treatment durations ranging from 1 h to 7 days. Some studies did not disclose the treatment duration or concentrations [25,26,36,38]. For animal studies, 0.5% *w*/*w* curcumin in the diet for 3 weeks was used with an oral route of administration [46], while intraperitoneal injection of curcumin (either 5 [46] or 50 mg/kg body weight/day [45]) for 2 to 4 weeks was employed.

Based on the OHAT critical appraisal tool, in vitro studies are inherently low risk in selection bias, performance bias, attrition/exclusion bias, and detection bias [50]. Nevertheless, all the included studies did not disclose blinding, even though it is not a common practice in reporting. The major issue is exposure characterisation, where most of the studies did not disclose the source [26,42], purity of curcumin [19,20,21,24,25,30,31,32,33,34,35,36,37,38,39,45], curcumin treatment duration, and tested concentrations [25,26,36,38,40] or leukemic cell concentration in treatment [25,26,36,38,40]. More than half of the included studies were not consistent with the treatment conditions [19,29,32,33,41] or did not disclose this information for some or all parameters [21,22,23,24,26,27,34,35,37,39,40,42,45]. Despite some quality issues, based on the tier grouping, all the included studies were ranked as Tier 1 because more than 50% of domains were classified as *Definitely* and/or *Probably low risk of bias* [20,22].

### 3.2. Curcumin Induces Cytotoxicity, Apoptosis, Autophagy, and Growth Arrest in ALL Cells While Sparing Non-Cancerous Cells

Curcumin was consistently reported to induce time- and/or concentration-dependent cytotoxicity and apoptosis in ALL cells [19,20,21,24,25,26,27,30,31,32,35,36,37,38,39,40,42,44,45]. Curcumin-treated cells demonstrated typical apoptotic features, including morphological alterations, DNA fragmentation, poly(ADP-ribose) polymerase (PARP) cleavage, mitochondrial dysfunction, cytochrome *c* release, BH3-interacting domain death agonist (Bid) cleavage, and downstream executioner caspases activation [19,20,21,24,25,30,32,35,36,39,40,42]. It also suppressed the colony-forming capacity of Bcr-Abl-positive SUP-B15 and RS4;11 cells [39], induced autophagy and autophagy-related cell death in SUP-B15 cells [37], and caused antiproliferation and growth arrest [25]. Curcumin induced G2/M arrest in CCRF-CEM, RS4;11 cells, REH cells [34,35], Jurkat T, and MOLT-4 cells [30] but G1 arrest in human EBV-related Burkitt lymphoma lymphoblast-like Raji cells [33] via upregulation of *CDKN2B* gene and downregulation of cyclin D1 and c-Myc. Despite these findings, the precise mechanisms underlying curcumin-mediated cell cycle arrest in ALL cells remain unclear.

Overall, the current evidence suggests that curcumin is more cytotoxic against the T-lineage of ALL cells than the B-lineage, with Jurkat T cells showing the greatest sensitivity. The reported IC_50_ values of 24 h curcumin treatment ranged from 4.22 μM and 36.5 μM in Jurkat T cells [32,40], decreasing to 2.89 μM upon 48 h treatment [32]. Similar time-dependent cytotoxicity of curcumin was observed in B-ALL REH (24 h IC_50_ of 21.81 μM; 48 h IC_50_ of 18.62 μM) and T-ALL MOLT-4 cells (24 h IC_50_ of 37.27 μM; 48 h IC_50_ of 23.72 μM) [32]. In T-ALL CCRF-CEM cells, the IC_50_ values were 6.49 μM [38], 8.68 μM [29], and 9.84 μM (unconfirmed treatment time) [26] upon 48 h treatment and reached 32.78 μM upon 72 h treatment [45]. Curcumin also induced cytotoxicity to other T-lineage ALL cells including doxorubicin-resistant CCRF-CEM cells (IC_50_: 21.04 µM) [38], B-ALL Raji cells (IC_50_: 20 µM) [33], T-ALL KOPT-K1 cells (IC_50_: 8.22 µM), T-ALL DND-41 cells (IC_50_: 13.255 µM), and TALL-1 cells (IC_50_: 6.33 µM) [42] upon 48 h treatment. It was also cytotoxic, but to a lesser degree, in B-ALL SUP-B15 cells, with IC_50_ values of 27.59–30.59 µM upon 72 h treatment [37,45].

Consistent with other cancer models, curcumin was selectively targeting ALL cells while sparing non-cancerous cells at the same tested concentrations. It was non-cytotoxic to non-cancerous cells, including rat skin fibroblasts, Chinese Hamster Ovarian CHO cells, mouse fibroblast L-929 cells, rat embryo fibroblast F111 cells, primary lymphocytes and hepatocytes (unknown origin, potentially rodent) [20], African green monkey kidney epithelial Vero cells [29], human corneal epithelial cells [20], and primary PBMCs from healthy donors [32,45]. However, Korwek et al. reported that curcumin induced apoptosis equally in both Jurkat T leukemic cells and primary T cells isolated from healthy donors [31].

### 3.3. Curcumin Activates Both Intrinsic and Extrinsic Apoptosis Pathways and Inhibits Oncogenic Signalling in ALL Cells

In in vitro ALL models, curcumin activates both intrinsic and extrinsic pathways of apoptosis, depending on cell type and experimental conditions. It was demonstrated to activate both caspase-8 and -9 in ALL cells with the decrease in procaspase and/or increase in cleaved caspases [21,24,39]. Specifically, curcumin induced the intrinsic pathway of apoptosis with the involvement of mitochondrial dysfunction, cytochrome *c* release, altered B cell lymphoma-2 (Bcl2)-associated X (Bax) to Bcl2 ratio, upregulation of mitochondrial pro-apoptotic proteins, and caspase-9 activation [19,21,24,32,39,45]. It also downregulated the inhibitors of caspases, including X-linked inhibitor of apoptosis protein (XIAP), cellular inhibitor of apoptosis protein (cIAP), and survivin [24,30,39], thus enhancing the susceptibility of leukemic cells to the intrinsic pathway of apoptosis. Mitochondrial membrane potential loss was observed in CCRF-CEM, HSB2, Jurkat T, and MOLT-4 cells upon 24 h treatment of 40 µM curcumin [24]. A higher concentration of curcumin (70 µM) caused mitochondrial membrane potential loss in Jurkat T cells as early as 4 h [40], while a lower concentration of 10 µM caused similar effects by 6 h [32]. Olicas-Aguirre et al. demonstrated that curcumin-induced mitochondrial dysfunction may result from mitochondrial uncoupling rather than mitochondrial calcium overload [40]. On the other hand, curcumin also activates the extrinsic pathway of apoptosis in ALL cells. It upregulated death receptors DR4 and DR5, induced Bid cleavage, and activated caspase-8 [21,24,31,41]. A non-toxic concentration of curcumin enhanced tumour necrosis factor (TNF)-related apoptosis-inducing ligand (TRAIL)-induced cytotoxicity and apoptosis in MOLT-4 cells and primary ALL cells from patients with B- and T-ALL [41]. The precise mechanism by which curcumin triggers mitochondrial dysfunction, releases mitochondrial proapoptotic proteins, and activates caspase-8 and 9 in ALL cells remains unclear.

Caspase activation in curcumin-treated ALL cells was found to be essential by using the pan-caspase inhibitor, z-VAD-FMK [24,30,32,39]. Pretreatment of z-VAD-FMK completely abrogated curcumin-mediated apoptosis, caspase-3 activation, and/or PARP cleavage in CCRF-CEM, Jurkat T, MOLT-4 cells [24,30], as well as in RS4;11 and SUP-B15 cells [39]. Gopal et al. demonstrated that caspase inhibition significantly but not completely protected Jurkat T cells from curcumin-induced cytotoxicity [32]. The IC_50_ of curcumin increased from 4.22 µM to 15 µM with z-VAD-FMK pretreatment [32]. Piwocka et al. demonstrated that a lower concentration and shorter pretreatment period of z-VAD-FMK (10 µM; 30 min) prevented ultraviolet-induced karyorrhexis but not curcumin-induced chromatin condensation in Jurkat T cells [19], suggesting suboptimal blocking of caspases. Curcumin-induced chromatin condensation occurred without caspase-3 activation, DNA fragmentation, and mitochondrial membrane potential loss [19], indicating caspase-independent cell death under short-term exposure to curcumin.

Curcumin exerts anti-leukemic effects in ALL cells by suppressing Janus kinase (JAK)/signal transducer and activator of transcription (STAT) [25,37,45] and phosphoinositol-3 kinase (PI3K)/AKT, followed by functional reactivation of Forkhead box O (FoxO)/glycogen synthase kinase 3β (GSK3β) [24,30,37,39,45] while activating the RAF/mitogen-activated protein kinase kinase (MEK)/extracellular signal-regulated kinase (ERK) pathway [37]. The PI3K/AKT and JAK/STAT pathway signalling cascades are highly associated with the leukemogenesis of ALL [52,53]. Curcumin induced dephosphorylation of AKT (inactivation), reducing downstream phosphorylation of FoxO and GSK3 (resulting as functional activation) in CCRF-CEM, Jurkat T and MOLT-4 cells [24,30,45], with similar effects observed in SUP-B15 and RS4;11 cells [39,45]. Guo et al. reported that curcumin suppressed the activation of AKT/mechanistic target of rapamycin (mTOR) but not phosphatase and Tensin homolog (pTEN) and 3-phosphoinositide-dependent protein kinase 1 (PDK1) in SUP-B15 cells and/or primary ALL cells isolated from the bone marrow of patients [37,45]. Curcumin also suppressed STAT5 activation in SUP-B15 cells in a time and concentration-dependent manner [45]. Concurrently, early and prolonged phosphorylation of RAF, MEK1/2, and ERK 1/2 was observed in curcumin-treated SUP-B15 cells, contributing to cell death rather than proliferation [37]. Further, chemical inhibition of MEK (by U0126) protected SUP-B15 cells from curcumin-mediated cytotoxicity and autophagy [37]. Similarly, it inhibited phosphorylation of JAK3, non-receptor tyrosine kinase 2 (TYK2), STAT3, and STAT5 in HTLV-1 transformed leukemic MT-2, HuT-102, and SLB-1 cells [25]. Curcumin also downregulated *BCR-ABL* mRNA in SUP-B15 cells [45] and inhibited nuclear factor κB (NF-κB) activation in REH cell apoptosis [36].

### 3.4. Curcumin Induces Oxidative Stress, DNA Damage, and Ceramide Accumulation as Upstream Apoptotic Signals

Curcumin exhibits mixed findings of oxidative stress and DNA damage induction in ALL cells, potentially depending on cell type and treatment conditions. Gopal et al. reported that curcumin induced reactive oxygen species (ROS) production and intracellular glutathione (GSH) depletion in REH, Jurkat T, and MOLT-4 cells, which could be prevented by N-acetyl cysteine (NAC) and GSH supplementation [32]. Similarly, curcumin induced ROS generation and chromosomal breaks in Raji cells [33], and it induced DNA damage with impaired DNA repair and Notch pathway suppression in KOPT-K1, DND41, and TALL-1 cells [42]. Parallelly, it caused ROS production, GSH depletion, DNA damage, and/or H2Ax (DNA damage sensing protein) activation in SUP-B15 and RS4;11 cells [39], as well as Jurkat T and MOLT-4 cells [30]. In contrast, Korwek et al. observed that 24 h curcumin exposure (up to 50 µM) did not induce significant DNA damage or DNA repair with no activation of H2Ax, ataxia-telangiectasia mutated protein (ATM), checkpoint kinase 2, and P53 in Jurkat T cells [31]. Similarly, Kong et al. also reported that curcumin (up to 200 µM) was not damaging pBR322 DNA plasmids in a cell-free system, unless in the presence of copper (II) ions (20 µM, a physiologically achievable concentration in serum) [29].

Interestingly, curcumin induced ceramide accumulation in ALL cell apoptosis, which is related to caspase activation and oxidative stress. Curcumin accumulated ceramide with a decrease in sphingomyelin synthase (SMS) activity but not ceramidase, sphingomyelinase, or glucosylceramide synthase [30]. Exogenous GSH prevented curcumin-induced apoptosis, SMS suppression, and ceramide production. Pan-caspase inhibition by z-VAD-FMK, as expected, could not block GSH depletion but surprisingly prevented curcumin-induced ceramide generation [30]. Pretreatment with buthionine sulphoximine (BSO; a GSH synthesis inhibitor) or D-609 (a SMS inhibitor) enhanced curcumin-mediated ceramide production and apoptosis [30].

### 3.5. Curcumin Downregulates WT1 and MDR1 and Upregulates CDK2NB Gene Expression

Curcumin downregulates Wilms’ tumour 1 (*WT1*) and multidrug resistance 1 (*MDR1*) gene expressions, which may contribute to its cytotoxicity in ALL cells. It reduced *WT1* mRNA expression in MOLT-4 cells [27] and primary ALL cells isolated from patient bone marrow [22], with greater effects in cells exhibiting high to moderate expression of *WT1* [22]. Interestingly, pure curcumin (95 to 99% purity), but not self-prepared or commercial-grade curcuminoid mixture (77% purity), significantly decreased *WT1* mRNA and protein levels in MOLT-4 cells [27]. Similarly, curcumin also downregulated *MDR1* in primary ALL cells isolated from patient bone marrow [23], with the strongest effects in cells with high to moderate *MDR1* expression. In other words, it was most effective in relapsed patients (60%), followed by newly diagnosed (56%), drug maintenance (50%), and completed treatment cases (43%) [23]. Parallel to this, it also significantly inhibited MDR1 (also known as P-gp) activity in doxorubicin-resistant, MDR1-overexpressed CCRF-CEM cells [38]. Nevertheless, the molecular mechanisms by which curcumin regulates these gene expression remain unclear.

Curcumin upregulated tumour suppressor gene in ALL cells, especially p15INK4b, a cyclin-dependent kinase inhibitor, partly through epigenetic regulation. *CDKN2B* (gene name for p15INK4b) is commonly hypermethylated in ALL [54,55]. A 72 h treatment of curcumin (5 and 10 µM) significantly induced G1 arrest in Raji cells via *CDKN2B* mRNA upregulation by reversing promoter hypermethylation and downregulating DNA methyltransferase 1 (*DNMT1*) mRNA [33]. At lower concentrations of 1 and 2 µM, it did not significantly upregulate *CDKN2B* and *CDH1* genes in CCRF-CEM cells with no apoptosis induction, despite an increasing trend in *CDKN2B* mRNA being observed [34]. Similarly, DNA pyrosequencing confirmed that curcumin did not significantly reverse DNA methylation at *CDKN2B* CpG sites under these low-concentration treatments [34]. Despite the lack of DNA hypomethylating activity, curcumin significantly upregulated Ten Eleven Translocation (*TET1/2/3*) enzymes concurrently, but not *DNMT1/3a/3b* [34]. Further studies are needed to confirm the mechanisms by which curcumin regulates DNA hypomethylation and gene expression in higher concentrations.

### 3.6. Curcumin Exhibits Limited Efficacy in ALL Xenograft Models

Preclinical in vivo studies of curcumin on ALL are scarce and show limited efficacy. Zunino et al. reported that oral supplementation (0.5% in diet for 3 weeks) and intraperitoneal injection (5 mg/kg body weight/day for 4 weeks) of curcumin did not reduce SEM leukemia cell growth in the blood of NOD/SCID mice [46]. Higher doses of curcumin injection (25–100 mg/kg body weight) were toxic and reduced the lifespan of mice; thus, Zunino et al. used a dose of 5 mg/kg that was not toxic. Nevertheless, curcumin, whether administered orally or intraperitoneally, did not improve the survival of mice compared to untreated controls [46]. In contrast, Guo et al. reported that a high dose of intraperitoneal curcumin (50 mg/kg body weight/day for 14 days) reduced SUP-B15 leukemic cell infiltration in the spleen and *BCR-ABL* mRNA in the bone marrow of immunosuppressed female BALB/c null mice [45]. Nevertheless, the safety profile of curcumin was not reported [45]. There is no report on the therapeutic effects of curcumin in patients with ALL.

## 4. Discussion

This systematic review highlights the anti-leukemic effects of curcumin in ALL, based on 26 selected in vitro and in vivo studies. Across studies, curcumin reduced ALL cell viability in a concentration- and time-dependent manner, mainly through intrinsic and extrinsic pathways of apoptosis with suppression of proliferative signalling pathways including PI3K/AKT and JAK/STAT, although some experimental variations lead to conflicting findings. For instance, Piwocka et al. reported that curcumin induced Jurkat T cell death, which is independent of DNA fragmentation, mitochondrial depolarisation, and caspase-3 activation [19]. This discrepancy might be due to the experimental design of transient exposure to curcumin (1 h treatment) followed by a 2 to 5 h curcumin-free recovery period. Most other studies assessed the cell death outcomes after 24 to 48 h of treatment. In addition, Piwocka et al. also defined cell death based on chromatin condensation from morphology observation [19], which is not always indicative of apoptosis [56]. Short and low concentration of z-VAD-FMK (10 µM; 30 min) in their study did not prevent curcumin-induced chromatin [19], while other studies used ≥50 µM and ≥1 h of z-VAD-FMK pretreatment [24,30,32,39]. Incomplete or suboptimal inhibition of caspases could account for these observed differences. In another study, Kong et al. demonstrated that curcumin did not cause DNA damage in a cell-free system unless in the presence of copper ions [32]. It is noted that copper ions can be present in cell culture media upon the enrichment of fetal bovine serum. On the other hand, Korwek et al. observed selective caspase-8 activation without detectable caspase-2 and -9 cleavage in curcumin-treated Jurkat T cells [31]. It is known that a variety of antibodies are available to evaluate caspase expression, either pro-, cleaved, or both forms [57,58]. The authors did not disclose the catalogue number of the antibodies used; hence, we were unable to confirm this. Band intensity analysis, however, indicated activation of procaspase-2 and -9, where the intensities of procaspase-2 and -9 were decreased along with curcumin treatment [31]. They also reported non-selective apoptosis induction in both Jurkat T cells and primary T lymphocytes from healthy donors, which may reflect biological and culture-related differences rather than experimental variation. Culture media [59,60,61] and serum batch-to-batch variation [62] are known to influence T lymphocyte metabolism and function. Nevertheless, the culture conditions for the isolated T lymphocytes were not reported.

Curcumin induces apoptosis in ALL cells primarily through oxidative stress and DNA damage, although the upstream molecular mechanisms remain unclear. Curcumin induced ROS generation and intracellular GSH depletion, leading to oxidative DNA damage and mitochondrial dysfunction [30,33,39,42]. This is consistent with previous findings where curcumin activated caspase-2 and/or -9 in ALL cells, which are known to be activated in DNA damage-mediated cell death [63,64]. At low concentrations, curcumin acts as an antioxidant, whereas at high concentrations, it behaves as a pro-oxidant in cancer cells [65]. In non-ALL models, curcumin has been shown to inhibit thioredoxin reductase and convert it to a NADPH oxidase to generate ROS [66,67,68]. Curcumin also induced oxidative stress through GSH depletion, which diminishes cellular defence against ROS. However, it is uncertain whether GSH depletion is mainly due to ROS scavenging or direct Michael addition with curcumin itself. GSH-conjugated curcumin has been detected in cell-free systems [69,70] and in human colorectal cancer Caco-2 cells [70]. In addition, NAC, which is membrane-permeable, can reduce intracellular radicals, whereas GSH cannot readily enter cells without the aid of specific membrane transporters [71,72,73]. Radicals like superoxide anion and hydroxyl radicals are highly reactive and are more likely to cause damage in situ rather than diffuse across the membrane to be neutralised by extracellular GSH. This explains why NAC and GSH supplementation, but not superoxide anion dismutase and catalase, prevented curcumin-induced GSH depletion in ALL cells [32]. We postulate that curcumin directly conjugates with thiol- or cysteine-rich molecules, reducing its availability to act on target pathways. Parallelly, curcumin binding to cysteamine in a cell-free thiol conjugation assay supports this hypothesis [43]. On the other hand, Kuttikrishnan et al. demonstrated that pan-caspase inhibition by z-VAD-FMK significantly reduced 24 h curcumin-mediated DNA damage in SUP-B15 and RS4;11 cells [39]. Thus, DNA damage can be secondary to cell death due to cell death-activated endo- and exonucleases rather than a primary initiating event. DNA integrity should therefore be assessed at earlier time points or in less toxic concentrations to clarify whether DNA damage precedes apoptosis.

Current evidence suggests that curcumin can inhibit oncogenic receptors and signalling pathways in ALL. In non-ALL cancer models, curcumin has been shown to inhibit hepatocyte growth factor and its receptor c-MET [74,75] and suppress human epidermal growth factor receptor 2 signalling [76,77]. In ALL, curcumin was reported to downregulate *BCR-ABL* mRNA expression in Bcr-Abl-positive SUP-B15 cells and corresponding xenograft in null mice [45]. Similar observations were reported in Bcr-Abl-positive chronic myeloid leukemia via competitive inhibition and epigenetic regulation [78,79]. Curcumin further synergised with imatinib (a first-line Bcr-Abl inhibitor) by suppressing AKT/mTOR signalling and downregulating the expression of *BCR-ABL* gene, an effect not seen with imatinib alone [45]. These findings suggest that curcumin may act through epigenetic or post-transcriptional regulation of Bcr-Abl expression. In addition, curcumin also inhibited JAK/STAT [25,37,45], PI3K/AKT [24,30,37,39,45], and NF-κB pathways [36] in ALL cells, which are known to be downstream canonical effectors of the Bcr-Abl signalling pathway [80,81,82]. Nevertheless, the evidence on Bcr-Abl signalling to date is mainly derived from studies of the P210 Bcr-Abl isoform in CML rather than the P190 isoform predominant in Philadelphia chromosome-positive ALL. Structurally, P190 Bcr-Abl protein lacks the Dbl-homology and Pleckstrin-homology domain with conserved ATP-binding pocket and major signalling pathways [83,84,85]. Consequently, the relevance of curcumin-mediated pathway inhibition in P190-driven ALL has not been directly established. In addition, direct molecular targets of curcumin in Bcr-Abl-negative ALL have yet to be identified, representing a critical gap in current knowledge.

**Figure 3:**
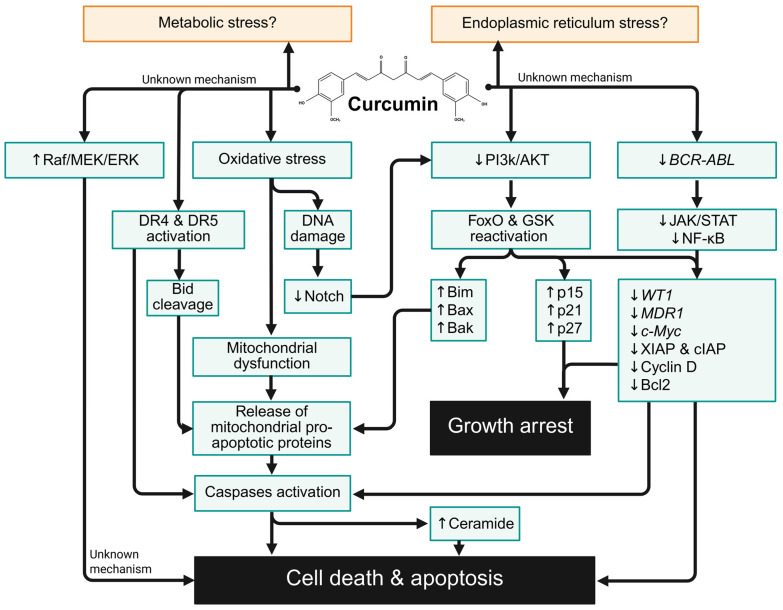
Summary of anti-leukemic properties of curcumin on ALL cells. ↑ Upregulation or increased level; ↓ downregulation or reduced level. Created in BioRender. Pang, K. (2026) https://BioRender.com/bdp2kkq, accessed on 25 December 2025.

Beyond oxidative stress and DNA-damaging activities, curcumin appears to target drug resistance-related molecular targets in reducing chemoresistance. Preclinical evidence demonstrates that curcumin downregulates *MDR1* and *WT1* genes and inhibits P-gp activity, highlighting its potential role as a chemosensitising agent in ALL [25,26,40]. It also prevented chemoresistance by inhibiting NF-κB activation, a pathway closely associated with chemoresistance in ALL cells [36]. Consistent with these findings, curcumin-mediated *WT1* downregulation has been reported across other leukemia subtypes, including acute and chronic myeloid leukemia [27,86,87,88,89,90,91]. Beyond ALL, it downregulated MDR gene in other leukemias, including mouse lymphocytic leukemia [92], acute myeloid leukemia [23,93], and chronic myeloid leukemia [23,94,95]. Curcumin also retained cytotoxic or chemosensitising activity in P-gp-overexpressed cervical cancer and breast cancer cells [28,96]. Moreover, structurally modified curcuminoids have demonstrated P-gp inhibitory activity as well [94,97,98,99]. Current preclinical evidence suggests that curcumin may function primarily as an adjunct to conventional chemotherapy rather than a single-target chemotherapeutic agent. Figure 3 summarizes the molecular mechanisms of anti-leukemic properties of curcumin on ALL cells.

Despite its potent anticancer activity in vitro, the clinical translation of curcumin is severely limited by its poor bioavailability. Curcumin is limited by poor water solubility, poor intestinal absorption, chemical instability, and rapid metabolism and excretion [100,101]. Consequently, blood curcumin levels are frequently undetectable or extremely low, with the majority of curcumin excreted in the feces [102,103]. In addition, curcumin is highly unstable under neutral aqueous solution, where about 90% of curcumin degraded non-enzymatically within 30 minutes [104]. This degradation is partially mitigated in culture media with serum due to stabilising interactions with biomolecules [105,106]. However, such protection is unlikely in an animal model. Curcumin failed to suppress leukemia cell growth or improve survival in NOD/SCID mice [46], which may be partially due to these limitations. Consistently, curcumin was rapidly metabolised into glucuronidated and sulfated conjugates in the plasma of mice as early as 1 h after administration [46]. From a medicinal chemistry standpoint, the β-diketone bridge of curcumin contributes to both chemical instability and metabolic susceptibility [107]. To overcome these limitations, chemical modification [108,109,110] and pharmaceutical strategies including nanoparticles, liposomes, and exosomes [105,111,112] have been widely used to improve the bioavailability of curcumin in acute myeloid leukemia (AML) treatment [113,114]. Nanoparticles, such as mesoporous silica and gold-based curcumin formulations, have exhibited increased cytotoxicity in cancer cells than free curcumin, while also improving its stability and bioavailability [105,114]. Liposomes, including curcumin modified with hyaluronan to target CD44, show higher binding affinity to CD44-overexpressing AML cells and improved cellular uptake [112,113]. Nevertheless, evidence supporting the efficacy of nanoformulated curcumin in ALL remains limited.

The structural–activity relationship of curcumin explains its biological activity, metabolic stability, and therapeutic potential [115], although it has not been systematically evaluated in the ALL model. The phenolic hydroxyl groups contribute to both antioxidant and anti-inflammatory activities as well as the key conjugation site during Phase II metabolism [116]. Chemical substitution of these hydroxyl groups has been shown to enhance metabolic stability and solubility. On the other hand, phenolic methoxy substituents are more strongly associated with anti-inflammatory and anticancer effects [115]. Consistent with this, curcumin, which contains two phenolic methoxy groups, demonstrates greater anticancer activity than demethoxycurcumin (single phenolic methoxy group) and bisdemethoxycurcumin (no phenolic methoxy group; Figure 1) in suppressing TNF-induced NF-κB activation [117] and inducing cytotoxicity in human glioblastoma LN229 and GBM8401 cells [118]. Similarly, pure curcumin (95–99% purity) significantly suppressed *WT1* mRNA and protein levels in MOLT-4 cells compared to a curcumin–demethoxycurcumin–bisdemethoxycurcumin mixture [27]. Synthetic curcumin analogues such as dimethoxycurcumin (Figure 1), which contains four phenolic methoxy groups, exhibited enhanced anticancer activity relative to curcumin, likely due to increased solubility, metabolic stability, cellular uptake, and bioavailability [108,109,119,120]. Supporting this, dimethoxycurcumin was proven less susceptible to liver microsomal enzymatic degradation and more potent in inducing DNA hypomethylation in ALL cells [34]. Similarly, polyphenols such as ferulic acid and ginger-derived compounds, including 6-gingerol, 6-shogaol, 10-gingerol, and 10-shogaol (Figure 1), share a common phenolic hydroxyl and methoxy structural feature and were reported to have similar anticancer, antioxidant, and anti-inflammatory mechanisms overlapping with those of curcumin [121,122,123,124,125,126,127,128,129,130]. These further highlight the importance of phenolic hydrocarbon structures in mediating curcumin’s biological activity.

In vivo and clinical evidence supporting the efficacy of curcumin in ALL remains scarce. In this systematic review, only two included articles reported animal data, and neither provided direct evidence that curcumin effectively eliminates ALL cells in animal models. Oral supplementation and intraperitoneal injection of curcumin failed to suppress the growth of SEM leukemia xenografts and did not improve survival outcomes in NOD/SCID mice [46]. A high dose of intraperitoneal injection of curcumin reduced SUP-B15 leukemic cells infiltration in the spleen of null mice [45]; however, the safety profile and reduction of leukemic cells in systemic circulation were not evaluated. To date, no published interventional studies have reported curcumin use in patients with ALL. Major clinical trial registries in the US (clinicaltrial.gov) and Europe (euclinicaltrials.eu) were searched to identify any ongoing or completed clinical trials evaluating curcumin in patients with ALL. We only identified a single registered trial conducted in Egypt between 2021 and 2024. This Phase II study (NCT05045443) [131] enrolled 30 paediatric patients aged 1 to 18 years with ALL and evaluated oral curcumin (500 mg turmeric-derived curcumin capsules, twice daily for one month), initiated during week 1 of the maintenance phase of chemotherapy. The primary outcome was the percentage of patients experiencing adverse events within four weeks. Although the trial status is listed as completed, no results have been published so far, leaving both the safety and therapeutic relevance of curcumin in patients with ALL unresolved. In addition, another related trial (NCT02100423) evaluated curcumin in combination with vitamin D, but it focused on patients with chronic lymphocytic leukemia or small lymphocytic lymphoma [132]. Similarly, it is completed, but the results remain unpublished. Despite these promising effects from in vitro studies, the optimal therapeutic dose of curcumin for ALL remains unclear. Evidence from studies in other cancers and non-cancer human conditions indicates that oral curcumin doses ranging from 20 mg/day up to 12 g/day are generally well tolerated, with minimal adverse effects [14,133,134]. Based on these findings, a similar dosing range could be considered in future ALL studies. 

This systematic review, like others, has some limitations. Methodological heterogeneity of included articles was found, including inconsistent treatment procedure, variable curcumin concentrations, differing treatment durations, and variable endpoint measurements. These inconsistencies may lead to errors in data interpretation and analysis. Future studies should be standardised in experimental procedures, including cell seeding and treatment conditions. Another limitation of this systematic review is the purity of the curcumin used. Most commercial-grade or extracted curcumin from turmeric powder contains around 30% of demethoxycurcumin and bisdemethoxycurcumin [22,23,27]. The composition of extracted curcuminoids varies greatly depending on the *Curcuma* species [108]. Only 3 out of 26 included studies used curcumin that was ≥90% pure [27,29,46]. In addition, demethoxycurcumin and bisdemethoxycurcumin share similar anticancer activities but with weaker potency than curcumin, including cytotoxicity [118,135], P-gp inhibition [28], and PI3K/AKT suppression [77]. This significantly hampers the interpretation of anti-leukemic effects of curcumin, as it is unclear whether the reported biological effects are from curcumin, curcuminoids or a mixture. Future studies should assess the anti-leukemic effects of pure curcumin and explicitly report the purity of the compound used. In terms of literature searching, we only considered primary studies written in English, and this search strategy may have overlooked grey literature and other relevant articles. To minimise this risk, we manually searched for more relevant articles from the reference list of included articles. Lastly, a meta-analysis was not conducted due to the heterogeneity in outcome measures across studies.

## 5. Conclusions

This systematic review provides an understanding of the selective cytotoxicity and apoptosis-inducing effects of curcumin in ALL cells, with T-lineage cells being more susceptible than B-lineage cells. Curcumin activates both intrinsic and extrinsic pathways of apoptosis, downregulates PI3K/AKT and JAK/STAT signalling pathways, downregulates *BCR-ABL*, *WT1* and *MDR1*, inhibits P-gp activity, and upregulates p15INK4b and RAF/MEK/ERK signalling. Upstream molecular mechanisms remain incompletely understood, but current evidence suggests the involvement of oxidative stress, DNA damage, and ceramide accumulation upon curcumin treatment. Despite promising in vitro results, in vivo evidence is limited, with only two ALL animal studies and no published clinical trials in patients with ALL. This highlights the need for Phase II/III clinical trials specifically targeting ALL, as most curcumin trials to date have examined other cancer types or non-ALL leukemias in general. Additional challenges for clinical translation include curcumin’s poor bioavailability and variability in curcuminoid composition. Further in-depth preclinical studies using standardised experimental protocols with well-designed clinical trials are essential to clarify curcumin’s therapeutic efficacy, optimal dosing, and safety profile as an anti-leukemic agent.

## Figures and Tables

**Table 1:** The anti-leukemic effects of curcumin on ALL based on in vitro, in vivo, and human studies.

Authors and Years	ALL Models	Curcumin	Treatment Condition	Findings	OHAT Tier
Piwocka et al. 1999 [19]	ALL cells: Human T-ALL Jurkat cells	Merck (Darmstadt, Germany), unknown catalogue number, unknown purity	50 μM for 1 h, followed by 2–5 h recovery without curcumin	Curcumin induced apoptosis in Jurkat cells with typical apoptotic features of chromatin condensation No involvement of DNA fragmentation, intracellular calcium changes, mitochondrial membrane potential changes, and caspase-3 activation Mitochondrial permeability transition pore opening was decreased rather than increased	1
Khar et al. 2001 [20]	ALL cells: Human T-ALL CCRF-CEM cells and Jurkat cells Non-cancerous cells: Rat skin fibroblasts, Chinese Hamster Ovarian CHO cells, mouse fibroblast L-929 cells, rat embryo fibroblast F111 cells, human corneal epithelial cells, primary lymphocytes and hepatocytes (unknown origin)	Sigma (St Louis, MO, USA), unknown catalogue number, unknown purity	10–75 μM for 24 h	Curcumin at 50 μM induced apoptosis (72 ± 3.4% for CCRF-CEM cells and 57 ± 4.8% for Jurkat cells) with DNA fragmentation Curcumin was non-cytotoxic to all tested non-cancerous cells	1
Duvoix et al. 2003 [21]	ALL cells: Jurkat cells	Sigma (Bornem, Belgium), unknown catalogue number, unknown purity	0–50 μM for 24 h	Curcumin was cytotoxic to Jurkat cells at ≥10 μM and caused caspase-8 and -9 activation, and subsequent Bid cleavage at 20 μM treatment for 24 h	1
Anuchapreeda et al. 2006 [22]	ALL cells: Primary ALL cells isolated from the bone marrow of patients with ALL at Maharaj Makon Chiang Mai Hospital, Chiang Mai, Thailand (*n* = 58; unknown basic demographic information)	Sigma (St Louis, MO, USA), unknown catalogue number, unknown purity	Ex vivo ALL cells were treated with 10 µM curcumin for 48 h	Curcumin reduced *WT1* gene expression, especially those cells with high to medium expression of *WT1*	1
Anuchapreeda et al. 2006 [23]	ALL cells: Primary ALL cells isolated from the bone marrow of patients with ALL at Maharaj Makon Chiang Mai Hospital, Chiang Mai, Thailand (*n* = 61; unknown basic demographic information)	Invitrogen™ Life Technology (Carlsbad, CA, USA), unknown catalogue number, 77% curcumin	Cells were treated with 10 µM curcumin for 48 h	Curcumin significantly reduced *MDR1* mRNA levels, especially those cells with high and moderate expression Curcumin-induced *MDR1* downregulation was affecting bone marrow cells from patients with relapsed ALL (60% of them), followed by newly diagnosed ALL (56%), drug maintenance (50%), and completed treatment (43%)	1
Hussain et al. 2006 [24]	ALL cells: Human T-ALL CCRF-CEM cells, Jurkat T cells, HSB2 cells, MOLT-4 cells	Sigma (St Louis, MO, USA), unknown catalogue number, unknown purity	10–80 µM for 24 h	Curcumin induced a concentration-dependent antiproliferation and apoptosis induction with PARP cleavage, caspases-8, -9, and -3 activation, downregulation of cIAP1, XIAP, and survivin, Bid cleavage, cytochrome *c* release, and mitochondrial membrane potential loss Curcumin induced dephosphorylation of AKT, FoxO transcription factor, and GSK3 in a concentration-dependent manner	1
Rajasingh et al. 2006 [25]	ALL cells: Human T-cell lymphotropic virus type-1 transformed T cell leukemic MT-2, HuT-102 and SLB-1 cells	Calbiochem (La Jolla, CA, USA), unknown catalogue number, unknown purity	1–25 μg/mL (2.7–67.5 μM) up to 48 h	Curcumin induced concentration-dependent growth arrest and apoptosis with chromatin condensation, membrane blebbing, and DNA fragmentation Curcumin inhibited phosphorylation of STAT3, STAT5, JAK3, and TYK2 in a concentration-dependent manner	1
Alaikov et al. 2007 [26]	ALL cells: CCRF-CEM cells	Unknown manufacturer, unknown catalogue number, unknown purity	Treatment time was unclear (48 h or 72 h)	Curcumin induced cytotoxicity (IC_50_ of 9.84 μM) in CCRF-CEM cells	1
Anuchapreeda et al. 2008 [27]	ALL cells: MOLT-4 cells	HPLC-extracted pure curcumin (95–99% purity based on their previous study [28]); Sigma (St Louis, MO, USA), unknown catalogue number, 77% purity	5–15 μM up to 3 days	Pure curcumin (0–15 μM) was cytotoxic to MOLT-4 cells. Pure curcumin, but not mixture or commercial grade curcumin (in mixture), reduced the *WT1* mRNA and its protein level in MOLT-4 cells	1
Kong et al. 2009 [29]	ALL cells: CCRF-CEM cells Non-cancerous cells: African green monkey kidney epithelial Vero cells	Sigma (St Louis, MO, USA), unknown catalogue number, 98% purity	Up to 20 μM for 48 h	Curcumin was more selectively cytotoxic to CCRF-CEM cells (IC_50_ of 8.68 μM) but not Vero cells (IC_50_ > 15 μM) Curcumin induced G2/M arrest in CCRF-CEM cells Curcumin was non-genotoxic up to 200 µM, with no DNA damage in cell-free pBR322 DNA plasmids Cytotoxicity and DNA-damaging properties of curcumin were enhanced in the presence of copper (II) ions, suggesting the role of reactive oxygen species	1
Kizhakkayil et al. 2012 [30]	ALL cells: Jurkat cells and MOLT-4 cells	Sigma (St Louis, MO, USA), unknown catalogue number, unknown purity	0–100 μM up to 24 h	Curcumin caused concentration- and time-dependent apoptosis induction with PARP cleavage, downregulation of XIAP, cIAP1, cIAP2, c-Myc, cyclin D1, and phosphorylated AKT levels, and GSH depletion in both Jurkat and MOLT-4 cells Curcumin induced a concentration-dependent accumulation of ceramide with a significant decrease in the activity of sphingomyelin synthase (SMS) but not ceramidase, sphingomyelinase, or glucosylceramide synthase Exogenous GSH prevented curcumin-induced ceramide generation and apoptosis Pretreatment with buthionine sulphoximine further enhanced curcumin’s activity in upregulating ceramide and apoptosis induction Pan-caspase inhibition (by z-VAD-FMK) prevented curcumin-induced ceramide generation and apoptosis induction but not GSH depletion Both exogenous GSH and z-VAD-FMK prevented curcumin-inhibited SMS activity SMS inhibition (by D-609) sensitised the Jurkat cells to further enhance curcumin-induced ceramide production and apoptosis	1
Korwek et al. 2013 [31]	ALL cells: Jurkat cells Non-cancerous cells: Human T cells from healthy donors (*n* = 9)	Cayman Chemical (Ann Arbor, MI, USA), unknown catalogue number, unknown purity	0–50 μM for 24 h	Curcumin did not selectively induce apoptosis in Jurkat or normal T cells in a concentration-dependent manner Curcumin activated caspase-8 and -3 but not caspase-2 and -9 Curcumin did not induce DNA damage, with no subsequent pathway activation	1
Gopal et al. 2014 [32]	ALL cell lines: Human B-ALL REH cells, Jurkat and MOLT-4 cells Non-cancerous cells: PBMCs from healthy donors	Sigma (St Louis, MO, USA), unknown catalogue number, unknown purity	2.5–50 μM for 24 h and 48 h	Curcumin induced concentration- and time-dependent cytotoxicity, antiproliferation, and apoptosis on REH (24 h IC_50_ of 21.81 μM; 48 h IC_50_ of 18.62 μM), Jurkat (24 h IC_50_ of 4.22 μM; 48 h IC_50_ of 2.89 μM), MOLT-4 cells (24 h IC_50_ of 37.27 μM; 48 h IC_50_ of 23.72 μM), with upregulation of ROS, intracellular GSH depletion, mitochondrial dysfunction, and caspase-9/-3 activation Curcumin had no apoptotic-inducing effect on PBMCs	1
Sharma et al. 2015 [33]	ALL cells: Human EBV-related Burkitt lymphoma lymphoblast-like Raji cells	Acros Organics (Fair Lawn, NJ, USA), unknown catalogue number, unknown purity	0–35 µM up to 6 days	Curcumin induced cytotoxicity (IC_50_ of 20 ± 2 µM), G1 arrest, and apoptosis with nuclear changes, apoptotic body formation, and DNA fragmentation Curcumin increased ROS production and caused chromosomal breaks Curcumin upregulated *CDKN2B* mRNA (p15INK4b) by reversing promoter methylation of p15INK4b and downregulated *DNMT1* mRNA	1
Hassan et al. 2015 [34]	ALL cells: CCRF-CEM cells	Sigma (Milwaukee, WI, USA), unknown catalogue number, unknown purity	1–10 μM for 72–168 h	Non-toxic concentration of curcumin (1 and 2 μM) induced G2/M arrest in CCRF-CEM cells Curcumin upregulated Ten Eleven Translocation (*TET1/2/3*) but not *DNMT1/3a/3b* mRNA At similar concentrations, curcumin did not induce apoptosis, upregulation of promoter-methylated genes, DNA hypomethylation, or reversal of global DNA methylation	1
Mishra et al. 2016 [35]	ALL cell lines: Human B-ALL RS4;11 and REH cells	Sigma (St. Louis, MO, USA), unknown catalogue number, unknown purity	10–100 μM for 24 h	Curcumin induced cytotoxicity, G2/M arrest, and apoptosis on RS4;11 and REH cells with PARP-1 cleavage	1
Pimentel-Gutiérrez et al. 2016 [36]	ALL cells: REH cells	Sigma (St Louis, MO, USA), unknown catalogue number, unknown purity	10–50 μM with unknown treatment duration	Curcumin induced concentration-dependent cytotoxicity and caspase-3 activation, with the reduction of NF-κB activation	1
Guo et al. 2018 [37]	ALL cells: SUP-B15 cells	Sigma, unknown catalogue number, unknown purity	30 μM for 4, 8, 24 and 72 h	Curcumin induced cytotoxicity (IC_50_ of 27.59 ± 7.06 µM), apoptosis, and autophagy in SUP-B15 cells, with upregulation of the RAF/MEK/ERK pathway but inhibition of Abl/STAT5 and AKT/mTOR pathways MEK inhibition (by U0126) protected the cells from curcumin-induced cytotoxicity and autophagy	1
Li et al. 2018 [38]	ALL cells: CCRF-CEM and doxorubicin-resistant CCRF-CEM (CEM/ADR 5000)	Fluka AG (Buchs, Switzerland), unknown catalogue number, unknown purity	Treatment up to 48 h, but unknown tested concentration range	Curcumin was cytotoxic to both wild-type and resistant CCRF-CEM cells with IC_50_ values of 6.49 ± 1.90 µM and 21.04 ± 2.24 µM, respectively Curcumin inhibited P-gp activity in CEM/ADR 5000 cells	1
Kuttikrishnan et al. 2019 [39]	ALL cells: B-ALL 697 cells, REH, SUP-B15, and RS4;11 cells	Sigma (St. Louis, MO, USA), unknown catalogue number, unknown purity	0–80 µM for 24 h	Curcumin induced cytotoxicity in all ALL cells. Curcumin induced apoptosis and suppressed colony-forming formation in SUP-B15 and RS4;11 cells, with DNA damage and H2Ax phosphorylation, ROS production, higher Bax/Bcl2 ratio, cytochrome c release, mitochondrial dysfunction, caspase-9/-3 activation, Akt suppression (GSK3/FoxO1 functional activation), lower cIAP and higher Bax/Bcl2 ratio NAC protected SUP-B15 cells from curcumin-induced ROS generation, DNA damage, caspase-3 activation, and apoptosis	1
Olivas-Aguirre et al. 2020 [40]	ALL cells: Jurkat cells	Sigma (St Louis, MO, USA), with the catalogue number C7727, ≥80% purity	0–200 µM for 24 h	Curcumin was cytotoxic to Jurkat cells (IC_50_ of 36.5 µM) and induced mitochondrial dysfunction, characterised by ROS production, loss of mitochondrial membrane potential, and mitochondrial uncoupling, without requiring mitochondrial calcium ion overload	1
Surapally et al. 2020 [41]	ALL cells: MOLT-4 cells Non-cancerous cells: PBMCs isolated from peripheral blood samples of patients with B-ALL (*n* = 20) and T-ALL (*n* = 2) from Apollo Cancer Specialty hospitals, Chennai, India	The source of curcumin was not disclosed	5 and 25 µM for 24 h	Curcumin (at 25 µM) increased the expression of Death Receptor DR4 and DR5 in MOLT-4 cells and T-ALL PBMCs Pretreatment of curcumin (at 5 µM) enhanced TRAIL-, IL2-TRAIL-, and IL2-TRAIL-peptide-induced cytotoxicity and apoptosis induction in MOLT-4 cells and PBMCs from patients with B- and T-ALL	1
Zhdanovskaya et al. 2022 [42]	ALL cells: Human T-ALL KOPT-K1, DND-41, and TALL-1 cells	Synthetic curcumin was prepared according to a previous study [43]	10 and 15 µM, up to 48 h	Curcumin was cytotoxic to KOPT-K1 (IC_50_ of 8.22 ± 0.817 µM), DND-41 (IC_50_ of 13.255 ± 2.269 µM), and TALL-1 cells (IC_50_ of 6.33 ± 0.884 µM) Curcumin induced DNA damage, ATM-mediated DNA repair, and apoptosis with PARP cleavage, caspase-3/7 activation, p27 upregulation, and decrease in *NOTCH1/3* mRNA and proteins in KOPT-K1, DND-41, and TALL1 cells Curcumin suppressed DNA repair enzymes (*BAP1*, *FEN1*, *RAD51*, *RNF8*, and *PCNA*) mRNA expression	1
Koszałka et al. 2022 [44]	ALL cells: MOLT-4 cells	Sigma (St Louis, MO, USA), catalogue number: C1386, ≥65% purity	6.1 and 12.2 µM, up to 72 h	Curcumin was cytotoxic to MOLT-4 cells	1
Guo et al. 2015 [45]	ALL cells: Human B-ALL SUP-B15 cells and CCRF-CEM cells, and primary ALL cells from bone marrow of patients with Philadelphia chromosome-positive ALL (*n* = 5), from West China Hospital of Sichuan University, China Non-cancerous cells: PBMCs from healthy donors	Sigma, unknown catalogue number, unknown purity	10–40 µM up to 5 days	Curcumin was cytotoxic and induced apoptosis on SUP-B15 (IC_50_ of 30.59 ± 7.06 µM) and CCRF-CEM cells (IC_50_ of 32.78 ± 5.32 µM) but not normal PBMCs (IC_50_ > 30 µM) Curcumin inhibited ABL/STAT5 and AKT/mTOR (GSK3β functional activation) but not Lyn, pTEN, and PDK1 with lower Bax/Bcl2 ratio in SUP-B15 cells and patient-derived primary ALL cells Curcumin downregulated Bcr/ABL mRNA expression in SUP-B15 cells	1
	Animal model: Immunosuppressed female BALB/c null mice with intravenous injection of SUP-B15 cells		Intraperitoneal injection of curcumin (50 mg/kg/day) 5 days per week for 14 days	Curcumin downregulated *BCR-ABL* gene expression in the bone marrow of mice Curcumin reduced the leukemic infiltration in the spleen	1
Zunino et al. 2013 [46]	Animal model: Non-obese diabetic/severe combined immunodeficient (NOD/SCID) mice with intravenous injection of human B-ALL SEM cells	Cayman Chemical Co. (Ann Arbor, MI, USA) with >90% purity (for oral) and from Axxora LLC (San Diego, CA, USA) with >98% purity (for injection)	Oral supplementation of curcumin (0.5% in diet) for 3 weeks Intraperitoneal injection of curcumin (5 mg/kg body weight/day) every other day for 4 weeks	Neither oral nor parenteral curcumin reduced the growth of SEM leukemia cells nor improved the survival of mice Curcumin and its metabolites were detected in the serum of mice	1

## Data Availability

This systematic review examined studies from publicly accessible databases, including PubMed, Scopus, Ovid MEDLINE, and Web of Science. No new data were created in this study.

## Supplementary Materials

The following supporting information can be downloaded at: https://www.mdpi.com/article/10.3390/biology15030258/s1, Table S1: PRISMA 2020 checklist [18]; Table S2: Search strategy used in this systematic review; Table S3: OHAT risk-of-bias tool for in vitro studies [20,21,22,23,24,25,26,27,29,30,31,32,33,34,35,36,37,38,39,40,41,42,44,45,46,47,48,49,50]; Table S4: OHAT risk-of-bias tool for animal studies [45,46].

## Abbreviations

The following abbreviations are used in this manuscript:

Abl	Abelson
AKT	Protein kinase A
ALL	Acute lymphoblastic leukemia
ATM	Ataxia-telangiectasia mutated protein
Bax	B cell lymphoma-2-associated X
Bcl2	B cell lymphoma-2
Bcr	breakpoint cluster region
Bid	BH3-interacting domain death agonist
BSO	Buthionine sulphoximine
cIAP	Cellular inhibitor of apoptosis protein
c-MET	Hepatocyte growth factor receptor
DNMT1	DNA methyltransferase 1
DR4	Death receptor 4
DR5	Death receptor 5
ERK	Extracellular signal-regulated kinase
FoxO	Forkhead box O
GSH	Glutathione
GSK3β	Glycogen synthase kinase 3β
HTLV	Human T-lymphotropic virus
IL2	Interleukin 2
JAK	Janus kinase
MDR1	Multidrug resistance 1
MEK	Mitogen-activated protein kinase
mTOR	Mechanistic target of rapamycin
NAC	N-acetyl cysteine
NF-κB	Nuclear factor κB
OHAT	Office of Health Assessment and Translation
PARP	Poly(ADP-ribose) polymerase
PDK1	3-phosphoinositide-dependent protein kinase 1
P-gp	P-glycoprotein
PI3K	Phosphoinositol-3 kinase
PRISMA	Preferred Reporting Items for Systematic Review and Meta-analyses
pTEN	Phosphatase and Tensin Homolog
ROS	Reactive oxygen species
SMS	Sphingomyelin synthase
STAT	Signal transducer and activator of transcription
TET	Ten Eleven Translocation
TNF	Tumour necrosis factor
TRAIL	Tumour necrosis factor-related apoptosis-inducing ligand
TYK2	Non-receptor tyrosine kinase 2
WT1	Wilms tumor1
XIAP	X-linked inhibitor of apoptosis protein
