# Supplementary material for: Anti-Leukemic Properties of Curcumin on Acute Lymphoblastic Leukemia: A Systematic Review
Source: Biology (Basel). 2026 Jan 30;15(3):258. doi: 10.3390/biology15030258 (PMC12897089; doi:10.3390/biology15030258)
Supplement: Supplementary file 1 [file biology-15-00258-s001.zip › biology-4103541-supplementary/biology-4103541-supplementary/Amended_Supplementary Table S3_OHAT risk-of-bias tool for in vitro studies.pdf]

Supplementary Table S3. OHAT risk-of-bias tool for in vitro studies

No.	OHAT in vitro studies domains	Included studies											
		Piwocka et al. [1]	Khar et al. [2]	Duvoix et al. [3]	Anuchapreeda et al. [4]	Anuchapreeda et al. [5]	Hussain et al. [6]	Rajasingh et al. [7]	Alaikov et al. [8]	Anuchapreeda et al. [9]	Kong et al. [10]	Kizhakkayil et al. [11]	Korwek et al. [12]
1	Was the administered dose or exposure level adequately randomized?	++	++	++	++	++	++	++	++	++	++	++	++
2	Was allocation to study groups adequately concealed?	++	++	++	++	++	++	++	++	++	++	++	++
3	Were experimental conditions identical across study groups?	++	++	++	++	++	++	++	++	++	++	++	++
4	Were research personnel blinded to the study group during the study?	-*	-*	-*	-*	-*	-*	-*	-*	-*	-*	-*	-*
5	Were outcome data complete without attrition or exclusion from analysis?	++	++	++	++	++	++	++	++	++	++	++	++
6	Can we be confident in the exposure characterization?	-**, ##	-#,##	-**, ##,###	-**,###	-**, #,###	- -**, #,##,###	-#,##,###	- -**, #,##,###	-**,###	-**, #	-#,##	-##
7	Can we be confident in the outcome assessment?	++	++	++	++	++	++	++	++	++	++	++	++
8	Were all measured outcomes reported?	++	++	++	++	++	++	++	++	++	++	++	++
9	Were there no other potential threats to internal validity?	++	++	++	++	++	++	++	++	++	++	++	++
**Overall appraisal: (Tier 1, Tier 2 or Tier 3)**		**1**	**1**	**1**	**1**	**1**	**1**	**1**	**1**	**1**	**1**	**1**	**1**

Note: + +, Definitely low risk; +, Probably low risk; -, Probably high risk; - -, Definitely high risk

* Did not mention about experimental blinding

** Not consistent or insufficient information of cell concentration across parameters

Used cell number instead of concentration in treatment

Did not disclose the purity of curcumin used

Did not disclose the stock preparation, storage and/or the maximal concentration of vehicle used

No.	OHAT in vitro studies domains (continue)	Included studies												
		Gopal et al. [13]	Sharma et al. [14]	Hassan et al. [15]	Mishra et al. [16]	Pimentel-Gutierrez et al. [17]	Guo et al. [18]	Li et al. [19]	Kuttikrishnan et al. [20]	Olivas-Aguirre et al. [21]	Surapally et al. [22]	Zhdanovskaya et al. [23]	Koszałka et al. [24]	Guo et al. [25]
1	Was administered dose or exposure level adequately randomized??	++	++	++	++	++	++	++	++	++	++	++	++	++
2	Was allocation to study groups adequately concealed?	++	++	++	++	++	++	++	++	++	++	++	++	++
3	Were experimental conditions identical across study groups?	++	++	++	++	++	++	++	++	++	++	++	++	++
4	Were research personnel blinded to the study group during the study?	-*	-*	-*	-*	-*	-*	-*	-*	-*	-*	-*	-*	-*
5	Were outcome data complete without attrition or exclusion from analysis?	++	++	++	++	++	++	++	++	++	++	++	++	++
6	Can we be confident in the exposure characterization?	- -**, #,##,###	- -**, #,##, ###	-**, ##	-**, #,##	- -#,##,###	-**, ##	- -#,##,###	-**,##	-**	-**, #,##	- -**,##,###	-#	-**, #,##
7	Can we be confident in the outcome assessment?	++	++	++	++	++	++	++	++	++	++	++	++	++
8	Were all measured outcomes reported?	++	++	++	++	++	++	++	++	++	++	++	++	++
9	Were there no other potential threats to internal validity?	++	++	++	++	++	++	++	++	++	++	++	++	++
**Overall appraisal: (Tier 1, Tier 2 or Tier 3)**		**1**	**1**	**1**	**1**	**1**	**1**	**1**	**1**	**1**	**1**	**1**	**1**	**1**

Note: + +, Definitely low risk; +, Probably low risk; -, Probably high risk; - -, Definitely high risk

* Did not mention about experimental blinding

** Not consistent or insufficient information of cell concentration across parameters

Used cell number instead of concentration in treatment

Did not disclose the purity of curcumin used

Did not disclose the information on stock preparation, storage and/or the maximal concentration of vehicle used

This tool was adapted from OHAT Risk of Bias Rating Tool for Human and Animal Studies based on previous studies [26-28]. Tier grouping was based on previous studies [27,29].

References:

1. Piwocka, K.; Zabłocki, K.; Wieckowski, M.R.; Skierski, J.; Feiga, I.; Szopa, J.; Drela, N.; Wojtczak, L.; Sikora, E. A novel apoptosis-like pathway, independent of mitochondria and caspases, induced by curcumin in human lymphoblastoid T (Jurkat) cells. *Exp Cell Res* **1999**, *249*, 299–307, doi:10.1006/excr.1999.4480.
2. Khar, A.; Ali, A.M.; Pardhasaradhi, B.V.; Varalakshmi, C.H.; Anjum, R.; Kumari, A.L. Induction of stress response renders human tumor cell lines resistant to curcumin-mediated apoptosis: role of reactive oxygen intermediates. *Cell Stress Chaperones* **2001**, *6*, 368–376, doi:10.1379/1466-1268(2001)006<0368:iosrrh>2.0.co;2.
3. Duvoix, A.; Morceau, F.; Schnekenburger, M.; Delhalle, S.; Galteau, M.M.; Dicato, M.; Diederich, M. Curcumin-induced cell death in two leukemia cell lines: K562 and Jurkat. *Ann N Y Acad Sci* **2003**, *1010*, 389–392, doi:10.1196/annals.1299.071.
4. Anuchapreeda, S.; Thanarattanakorn, P.; Sittipreechacharn, S.; Chanarat, P.; Limtrakul, P. Curcumin inhibits WT1 gene expression in human leukemic K562 cells. *Acta Pharmacol Sin* **2006**, *27*, 360–366, doi:10.1111/j.1745-7254.2006.00291.x.
5. Anuchapreeda, S.; Thanarattanakorn, P.; Sittipreechacharn, S.; Tima, S.; Chanarat, P.; Limtrakul, P. Inhibitory effect of curcumin on MDR1 gene expression in patient leukemic cells. *Arch Pharm Res* **2006**, *29*, 866–873, doi:10.1007/bf02973907.
6. Hussain, A.R.; Al-Rasheed, M.; Manogaran, P.S.; Al-Hussein, K.A.; Platanias, L.C.; Al Kuraya, K.; Uddin, S. Curcumin induces apoptosis via inhibition of PI3'-kinase/AKT pathway in acute T cell leukemias. *Apoptosis* **2006**, *11*, 245–254, doi:10.1007/s10495-006-3392-3.
7. Rajasingh, J.; Raikwar, H.P.; Muthian, G.; Johnson, C.; Bright, J.J. Curcumin induces growth-arrest and apoptosis in association with the inhibition of constitutively active JAK-STAT pathway in T cell leukemia. *Biochem Biophys Res Commun* **2006**, *340*, 359–368, doi:10.1016/j.bbrc.2005.12.014.
8. Alaikov, T.; Konstantinov, S.M.; Tzanova, T.; Dinev, K.; Topashka-Ancheva, M.; Berger, M.R. Antineoplastic and anticlastogenic properties of curcumin. *Ann N Y Acad Sci* **2007**, *1095*, 355–370, doi:10.1196/annals.1397.039.
9. Anuchapreeda, S.; Tima, S.; Duangrat, C.; Limtrakul, P. Effect of pure curcumin, demethoxycurcumin, and bisdemethoxycurcumin on WT1 gene expression in leukemic cell lines. *Cancer Chemother Pharmacol* **2008**, *62*, 585–594, doi:10.1007/s00280-007-0642-1.
10. Kong, Y.; Ma, W.; Liu, X.; Zu, Y.; Fu, Y.; Wu, N.; Liang, L.; Yao, L.; Efferth, T. Cytotoxic activity of curcumin towards CCRF-CEM leukemia cells and its effect on DNA damage. *Molecules* **2009**, *14*, 5328–5338, doi:10.3390/molecules14125328.
11. Kizhakkayil, J.; Thayyullathil, F.; Chathoth, S.; Hago, A.; Patel, M.; Galadari, S. Glutathione regulates caspase-dependent ceramide production and curcumin-induced apoptosis in human leukemic cells. *Free Radic Biol Med* **2012**, *52*, 1854–1864, doi:10.1016/j.freeradbiomed.2012.02.026.
12. Korwek, Z.; Bielak-Zmijewska, A.; Mosieniak, G.; Alster, O.; Moreno-Villanueva, M.; Burkle, A.; Sikora, E. DNA damage-independent apoptosis induced by curcumin in normal resting human T cells and leukaemic Jurkat cells. *Mutagenesis* **2013**, *28*, 411–416, doi:10.1093/mutage/get017.
13. Gopal, P.K.; Paul, M.; Paul, S. Curcumin induces caspase mediated apoptosis in JURKAT cells by disrupting the redox balance. *Asian Pac J Cancer Prev* **2014**, *15*, 93–100, doi:10.7314/apjcp.2014.15.1.93.
14. Sharma, V.; Jha, A.K.; Kumar, A.; Bhatnagar, A.; Narayan, G.; Kaur, J. Curcumin-Mediated Reversal of p15 Gene Promoter Methylation: Implication in Anti-Neoplastic Action against Acute Lymphoid Leukaemia Cell Line. *Folia Biol (Praha)* **2015**, *61*, 81–89.
15. Hassan, H.E.; Carlson, S.; Abdallah, I.; Buttolph, T.; Glass, K.C.; Fandy, T.E. Curcumin and dimethoxycurcumin induced epigenetic changes in leukemia cells. *Pharm Res* **2015**, *32*, 863–875, doi:10.1007/s11095-014-1502-4.
16. Mishra, D.; Singh, S.; Narayan, G. Curcumin Induces Apoptosis in Pre-B Acute Lymphoblastic Leukemia Cell Lines Via PARP-1 Cleavage. *Asian Pac J Cancer Prev* **2016**, *17*, 3865–3869.
17. Pimentel-Gutiérrez, H.J.; Bobadilla-Morales, L.; Barba-Barba, C.C.; Ortega-De-La-Torre, C.; Sánchez-Zubieta, F.A.; Corona-Rivera, J.R.; González-Quezada, B.A.; Armendáriz-Borunda, J.S.; Silva-Cruz, R.; Corona-Rivera, A. Curcumin potentiates the effect of chemotherapy against acute lymphoblastic leukemia cells via downregulation of NF-κB. *Oncol Lett* **2016**, *12*, 4117–4124, doi:10.3892/ol.2016.5217.

18. Guo, Y.; Shan, Q.Q.; Gong, P.Y.; Wang, S.C. The autophagy induced by curcumin via MEK/ERK pathway plays an early anti-leukemia role in human Philadelphia chromosome-positive acute lymphoblastic leukemia SUP-B15 cells. *J Cancer Res Ther* **2018**, *14*, S125–s131, doi:10.4103/0973-1482.172111.
19. Li, H.; Krstin, S.; Wink, M. Modulation of multidrug resistant in cancer cells by EGCG, tannic acid and curcumin. *Phytomedicine* **2018**, *50*, 213–222, doi:10.1016/j.phymed.2018.09.169.
20. Kuttikrishnan, S.; Siveen, K.S.; Prabhu, K.S.; Khan, A.Q.; Ahmed, E.I.; Akhtar, S.; Ali, T.A.; Merhi, M.; Dermime, S.; Steinhoff, M.; et al. Curcumin Induces Apoptotic Cell Death via Inhibition of PI3-Kinase/AKT Pathway in B-Precursor Acute Lymphoblastic Leukemia. *Front Oncol* **2019**, *9*, 484, doi:10.3389/fonc.2019.00484.
21. Olivas-Aguirre, M.; Torres-López, L.; Pottosin, I.; Dobrovinskaya, O. Phenolic Compounds Cannabidiol, Curcumin and Quercetin Cause Mitochondrial Dysfunction and Suppress Acute Lymphoblastic Leukemia Cells. *Int J Mol Sci* **2020**, *22*, doi:10.3390/ijms22010204.
22. Surapally, S.; Jayaprakasam, M.; Verma, R.S. Curcumin augments therapeutic efficacy of TRAIL-based immunotoxins in leukemia. *Pharmacol Rep* **2020**, *72*, 1032–1046, doi:10.1007/s43440-020-00073-7.
23. Zhdanovskaya, N.; Lazzari, S.; Caprioglio, D.; Firrincieli, M.; Maioli, C.; Pace, E.; Imperio, D.; Talora, C.; Bellavia, D.; Checquolo, S.; et al. Identification of a Novel Curcumin Derivative Influencing Notch Pathway and DNA Damage as a Potential Therapeutic Agent in T-ALL. *Cancers (Basel)* **2022**, *14*, doi:10.3390/cancers14235772.
24. Koszałka, P.; Stasiłojć, G.; Miękus-Purwin, N.; Niedźwiecki, M.; Purwin, M.; Grabowski, S.; Bączek, T. The Cooperative Anti-Neoplastic Activity of Polyphenolic Phytochemicals on Human T-Cell Acute Lymphoblastic Leukemia Cell Line MOLT-4 In Vitro. *Int J Mol Sci* **2022**, *23*, doi:10.3390/ijms23094753.
25. Guo, Y.; Li, Y.; Shan, Q.; He, G.; Lin, J.; Gong, Y. Curcumin potentiates the anti-leukemia effects of imatinib by downregulation of the AKT/mTOR pathway and BCR/ABL gene expression in Ph+ acute lymphoblastic leukemia. *Int J Biochem Cell Biol* **2015**, *65*, 1–11, doi:10.1016/j.biocel.2015.05.003.
26. Hameete, B.C.; Plösch, T.; Hogenkamp, A.; Groenink, L. A systematic review and risk of bias analysis of in vitro studies on trophoblast response to immunological triggers. *Placenta* **2025**, *166*, 164–175, doi:https://doi.org/10.1016/j.placenta.2024.10.010.
27. Pang, K.-L.; Mai, C.-W.; Chin, K.-Y. Molecular Mechanism of Tocotrienol-Mediated Anticancer Properties: A Systematic Review of the Involvement of Endoplasmic Reticulum Stress and Unfolded Protein Response. *Nutrients* **2023**, *15*, 1854.
28. National Toxicology Program. Risk of Bias Tool. Available online: https://ntp.niehs.nih.gov/research/assessments/noncancer/riskbias (accessed on September 20 2025).
29. Romeo, S.; Zeni, O.; Sannino, A.; Lagorio, S.; Biffoni, M.; Scarfì, M.R. Genotoxicity of radiofrequency electromagnetic fields: Protocol for a systematic review of in vitro studies. *Environment International* **2021**, *148*, 106386, doi:https://doi.org/10.1016/j.envint.2021.106386.
