# Supplementary material for: Anti-Leukemic Properties of Curcumin on Acute Lymphoblastic Leukemia: A Systematic Review
Source: Biology (Basel). 2026 Jan 30;15(3):258. doi: 10.3390/biology15030258 (PMC12897089; doi:10.3390/biology15030258)
Supplement: Supplementary file 1 [file biology-15-00258-s001.zip › biology-4103541-supplementary/biology-4103541-supplementary/Amended_Supplementary Table S4_OHAT risk-of-bias tool for animal studies.pdf]

Supplementary Table S4. OHAT risk-of-bias tool for animal studies

No.	OHAT for animal studies domains	Included studies	
		Guo et al. 2015 [1]	Zunino et al. 2013 [2]
1	Was administered dose or exposure level adequately randomized?	- $	++
2	Was allocation to study groups adequately concealed?	++	++
3	Did the study design or analysis account for important confounding and modifying variables?	++	++
4	Were experimental conditions identical across study groups?	++	++
5	Were research personnel blinded to the study group during the study?	- $	- $
6	Were outcome data complete without attrition or exclusion from analysis?	++	++
7	Can we be confident in the exposure characterization?	- $$	++
8	Can we be confident in the outcome assessment?	- θ	++
9	Were all measured outcomes reported?	++	++
10	Were there no other potential threats to internal validity?	+ θθ	++
**Overall appraisal: (Tier 1, Tier 2 or Tier 3)**		**1**	**1**

Note:

+ +, Definitely low risk; +, Probably low risk; -, Probably high risk; - -, Definitely high risk

$ No direct evidence

$$ Did not disclose the purity of curcumin used

θ No quantitative analysis

θθ No dietary information/control of curcumin

References:

1. Guo, Y.; Li, Y.; Shan, Q.; He, G.; Lin, J.; Gong, Y. Curcumin potentiates the anti-leukemia effects of imatinib by downregulation of the AKT/mTOR pathway and BCR/ABL gene expression in Ph+ acute lymphoblastic leukemia. *Int J Biochem Cell Biol* **2015**, *65*, 1–11, doi:10.1016/j.biocel.2015.05.003.
2. Zunino, S.J.; Storms, D.H.; Newman, J.W.; Pedersen, T.L.; Keen, C.L.; Ducore, J.M. Oral or parenteral administration of curcumin does not prevent the growth of high-risk t(4;11) acute lymphoblastic leukemia cells engrafted into a NOD/SCID mouse model. *Int J Oncol* **2013**, *42*, 741–748, doi:10.3892/ijo.2012.1734.
