# Supplementary material for: Anti-Leukemic Properties of Curcumin on Acute Lymphoblastic Leukemia: A Systematic Review
Source: Biology (Basel). 2026 Jan 30;15(3):258. doi: 10.3390/biology15030258 (PMC12897089; doi:10.3390/biology15030258)
Supplement: Supplementary file 1 [file biology-15-00258-s001.zip › biology-4103541-supplementary/biology-4103541-supplementary/Supplementary Table S2_Search strategy used in this systematic review.pdf]

Supplementary Table S2. Search strategy used in this systematic review

Database	Search strategy
PubMed	((curcumin) OR ("natural yellow 3") OR (diferuloylmethane) OR ("turmeric yellow")) AND ((leukaem*) OR (leukem*))
WoS	((curcumin) OR ("natural yellow 3") OR (diferuloylmethane) OR ("turmeric yellow")) AND ((leukaem*) OR (leukem*)); in all field
Scopus	((curcumin) OR ("natural yellow 3") OR (diferuloylmethane) OR ("turmeric yellow")) AND ((leukaem*) OR (leukem*)); Title, Abstract, Keywords only; limited to English and Article type.
Ovid MEDLINE	((curcumin) OR ("natural yellow 3") OR (diferuloylmethane) OR ("turmeric yellow")) AND ((leukaem*) OR (leukem*)); in all field.
